# Case Report: Fetal cardiac rhabdomyoma caused by TSC1 mutation

**DOI:** 10.3389/fped.2025.1681439

**Published:** 2025-10-10

**Authors:** Xueqin Feng, Qinnggui Ren, Haihong Li, Xinying Liu, Lijuan Li

**Affiliations:** ^1^ Department of Obstetrics, Affiliated Hospital of Jining Medical University, Jining, China; ^2^ Department of Mammary Gland Surgery, Affiliated Hospital of Jining Medical University, Jining, China; ^3^ College of Clinical Medicine for Obstetrics & Gynecology and Pediatrics, Fujian Medical University, Fuzhou, China

**Keywords:** fetal rhabdomyoma, tuberous sclerosis complex (TSC), TSC1 mutation, prenatal diagnosis, whole-exome sequencing

## Abstract

Fetal rhabdomyoma is a rare benign cardiac tumor that primarily occurs during the fetal or neonatal period and is often associated with Tuberous Sclerosis Complex (TSC). It is most commonly found in the heart (particularly in the ventricles or interventricular septum) but can also occur in other locations such as the head and neck. It may be accompanied by arrhythmias (e.g., supraventricular tachycardia), pericardial effusion, or fetal hydrops. Larger tumors can lead to blood flow obstruction, heart failure, or sudden death. In this case, prenatal ultrasound at 22 weeks of gestation suggested a “possible fetal left ventricular rhabdomyoma” in the proband. The parents were advised to undergo prenatal diagnosis but declined and opted for induced labor. Whole-exome sequencing (familybased) revealed a heterozygous TSC1 mutation in the proband, while both parents exhibited wildtype genotypes. This case report presents an instance of fetal cardiac rhabdomyoma induced by a heterozygous TSC1 mutation, providing valuable insights for the early diagnosis and management of intrauterine fetal cardiac developmental abnormalities.

## 1 Introduction

Fetal rhabdomyoma, a rare benign tumor, despite its low incidence during the fetal period, has emerged as a critical focus of prenatal diagnosis, given its potential to significantly impact fetal cardiovascular and overall development (1). The majority of these tumors originate from the myocardium, with 80%–90% located in the heart (predominantly in the ventricular wall or interventricular septum), while a smaller proportion are found in the head and neck regions. When visualized on prenatal ultrasound, fetal rhabdomyoma typically presents as single or multiple hyperechoic nodules within the myocardium, exhibiting substantial inter-individual variability in size, number, and growth rate. Large or rapidly growing tumors may lead to severe complications, including compression of cardiac structures, which can result in blood flow obstruction and heart failure, disruption of the electrical conduction system causing arrhythmias, and, in some instances, the presence of pericardial effusion or fetal hydrops. It is estimated that approximately 75% of fetal cardiac rhabdomyomas are associated with Tuberous Sclerosis Complex (TSC), an autosomal dominant neurocutaneous syndrome (2). Activation of the mTOR pathway, which is triggered by mutations in TSC1 or TSC2 genes, results in abnormal myocardial proliferation. Cardiac rhabdomyoma frequently represents the earliest and most characteristic manifestation of TSC during the fetal period (1).

In this article, a case is presented in which a hyperechoic mass in the left ventricle was identified via ultrasound at 22 weeks of gestation. Subsequent genetic testing following induced labor confirmed a heterozygous mutation in the TSC1 gene, suggesting that the condition might be attributed to a *de novo* mutation in the absence of a family history. This finding underscores the critical role of prenatal genetic testing in the diagnosis and prognosis assessment of such cases.

## 2 Case report

### 2.1 Clinical data of the patient

A 25-year-old nulliparous female (G1P0) was admitted to our hospital in March 2025, a case with a 24 + 3-week pregnancy and a decision for medically indicated labor induction admitted to our clinic. Regular prenatal care was maintained throughout gestation. In the first trimester, she received “Baotai Pill” and dydrogesterone for 1 month due to perigestational sac fluid accumulation. Nuchal translucency (NT) measured 1.2 mm, and non-invasive prenatal testing (NIPT) showed low risk for common aneuploidies (trisomy 21, 18, and 13), which does not equate to a confirmed normal karyotype. At 21 weeks, an external hospital B-ultrasound detected “punctate hyperechogenicity in the fetal left ventricle” with a recommendation for temporary observation. At 22 weeks, color Doppler ultrasound at another local medical facility revealed “a hyperechoic mass in the fetal left ventricle (suspicious for rhabdomyoma) with bilateral ventricular punctate hyperechogenicities”, prompting referral to a tertiary maternal-fetal medicine center for further assessment and genetic counseling. Subsequent fetal echocardiographyidentified a 4.6 mm × 5.1 mm × 3.3 mm hyperechoic nodule near the fetal left ventricular apex (suspicious for rhabdomyoma), with amniocentesis recommended but declined due to concerns about procedure-related miscarriage risk. Further consultation with a multidisciplinary team reaffirmed the need for prenatal diagnosis; however, the patient and family opted for induced labor and presented with relevant documentation.

On admission, she was asymptomatic (no abdominal pain, vaginal bleeding, or rupture of membranes) with normal perceived fetal movements. The admission diagnosis was “24 + 3 weeks of gestation with fetal cardiac developmental abnormalities”. Her general condition remained good throughout pregnancy, with normal mental status, appetite, sleep, and bowel function, and a total weight gain of ∼10 kg. She was classified as “green” risk via the maternal pregnancy five-color assessment system and held a maternal-child health manual. No family history of genetic or infectious diseases was noted. Her spouse was healthy, with no exposure to adverse factors during gestation.

### 2.2 Admission examinations and genetic testing results

**Figure 1:**
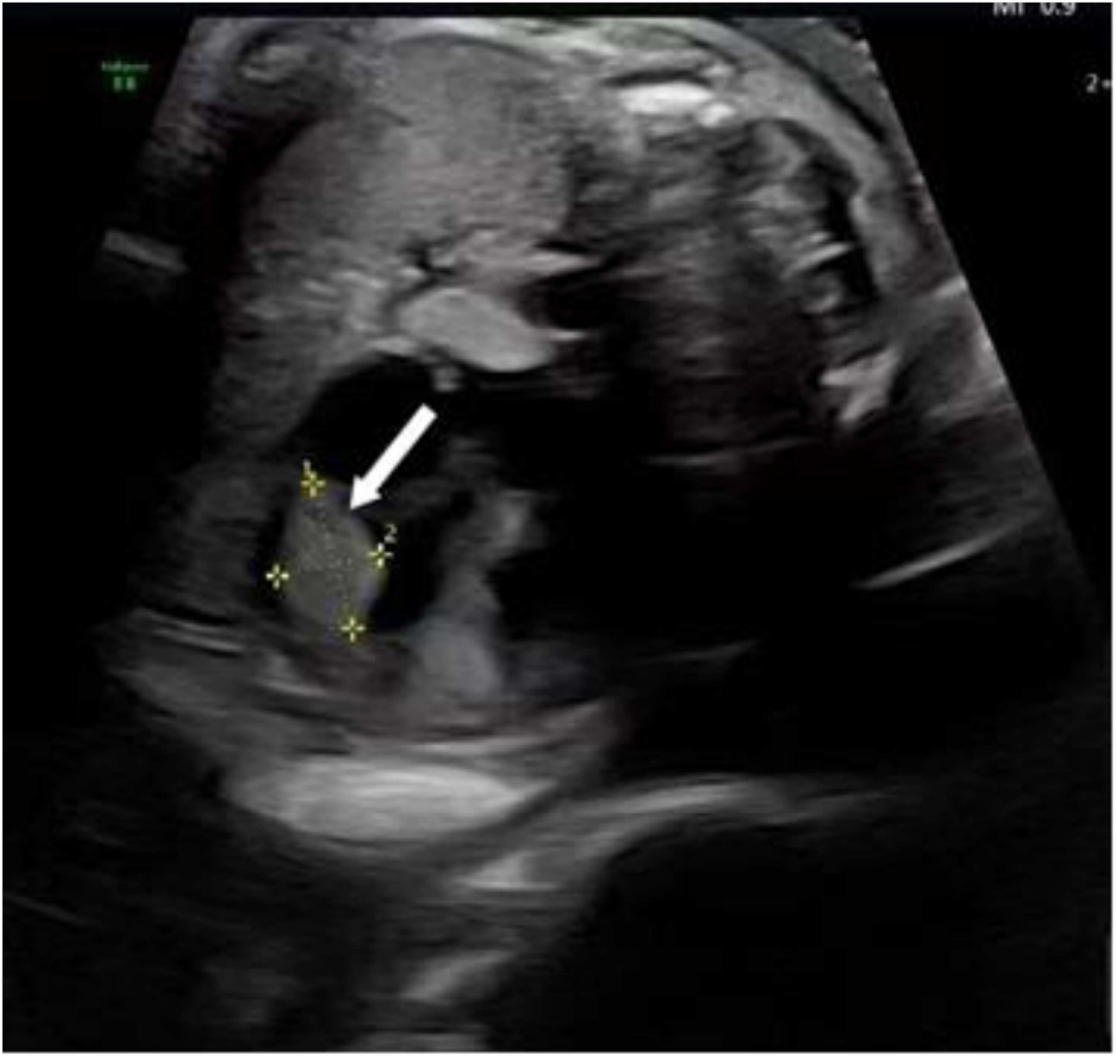
Ultrasonography reconfirmed rhabdomyoma near the apex of the fetal left ventricle, as as indicated by the arrow.

Following admission, the patient underwent further evaluations, with ultrasonography reconfirming a hyperechoic nodule near the apex of the fetal left ventricle, consistent with rhabdomyoma (Figure 1). Fetal echocardiography confirmed a 4.6 mm × 5.1 mm × 3.3 mm cardiac rhabdomyoma in the left ventricular free wall. No tricuspid or mitral valve insufficiency was detected, and cardiac output was within the normal range for gestational age. There was no evidence of fetal hydrops (ascites, pleural effusion, or skin thickening) after obtaining informed consent from the patient and her family, induction of labor was performed on March 6, 2025, via ultrasound-guided intra-amniotic injection of rivanol combined with mifepristone. Immediately following expulsion of the stillbirth, fetal skin tissue and peripheral blood samples from both parents were collected for genetic analyses, including family-based whole-exome sequencing and chromosome copy number variation (CNV) testing.

Genetic analysis revealed a heterozygous variant in the fetal TSC1 gene (NM_000368.4:c.1498C>T, p.Arg500). Localized to exon 15 of TSC1, this variant results in premature termination of translation of the encoded hamartin protein, consistent with a nonsense mutation. Based on the American College of Medical Genetics and Genomics (ACMG) guidelines-incorporating variant type and population frequency-the variant was classified as likely pathogenic (PVS1 + PM2). Specifically, PVS1 (strong pathogenic evidence) applies because the nonsense mutation aligns with the loss-of-function (LOF) mechanism underlying TSC1-related pathogenesis; PM2 (moderate evidence) applies as the variant is absent from databases such as 1,000 Genomes, ESP6500, ExAC, and GnomAD, indicating rarity in the general population.

The TSC1 p.Arg500 nonsense mutation identified in this case results in a truncated TSC1 protein, which is predicted to disrupt mTOR pathway regulation (ClinVar Accession: VCV000XXXX.1). According to the LOVD database, similar TSC1 nonsense mutations (e.g., p.Trp499Ter, p.Arg501Ter) are associated with a higher risk of early-onset tuberous sclerosis complex (TSC) manifestations, including cardiac rhabdomyomas and cortical tubers. However, phenotypic variability exists, with some patients presenting only mild neurological symptoms in childhood (2).

**Figure 2:**
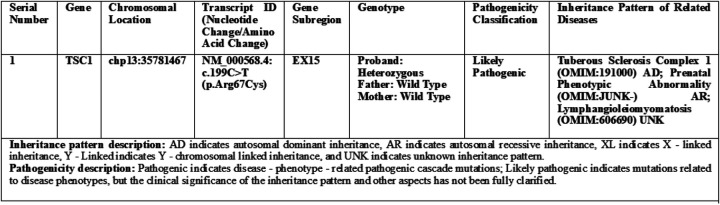
Whole exome sequencing test (1) report (family-based).

Family segregation analysis confirmed that both parents carried wild-type TSC1 alleles, confirming the fetal variant as a *de novo* mutation-neither inherited from the family nor attributable to germline mosaicism. Additionally, chromosomal analysis via whole-genome single nucleotide polymorphism microarray (SNP array) identified no abnormal copy number variations, excluding chromosomal disorders (Figure 2).

## 3 Discussion

### 3.1 Correlation between cardiac rhabdomyoma and TSC

Cardiac rhabdomyoma represents the most common benign cardiac tumor in the fetal period, with approximately 75% of cases associated with Tuberous Sclerosis Complex (TSC) (3). TSC is an autosomal dominant neurocutaneous disorder caused by mutations in TSC1 or TSC2, clinically characterized by the development of hamartomas across multiple organ systems. Its incidence among newborns ranges from 1 in 6,000 to 1 in 1,000 (4). TSC is a neurocutaneous syndrome characterized by multisystem hamartomas, with neurological manifestations being a major source of morbidity (5). Prenatal brain MRI can detect cortical tubers or subependymal nodules, which are early indicators of neurological involvement. For surviving fetuses with TSC, the risk of epilepsy (typically developing before the age of 2) and neurodevelopmental delays increases with the number of cortical tubers (6). Therefore, long-term neurological follow-up, including developmental assessments and EEG monitoring, is essential to enable early intervention. Cardiac rhabdomyoma.

As a specific fetal manifestation of TSC, with the pathogenesis of cardiac rhabdomyoma involving TSC1 mutations that induce aberrant activation of the mammalian target of rapamycin (mTOR) pathway, resulting in abnormal myocardial cell proliferation. Approximately 50%–70% of fetuses with TSC develop cardiac rhabdomyoma, whereas involvement of organs such as the brain and kidneys may emerge progressively after birth as development proceeds (7, 8). Consequently, prenatal diagnosis of cardiac rhabdomyoma is critical for assessing fetal prognosis and guiding delivery planning.

### 3.2 Prenatal diagnosis strategy

**Figure 3:**
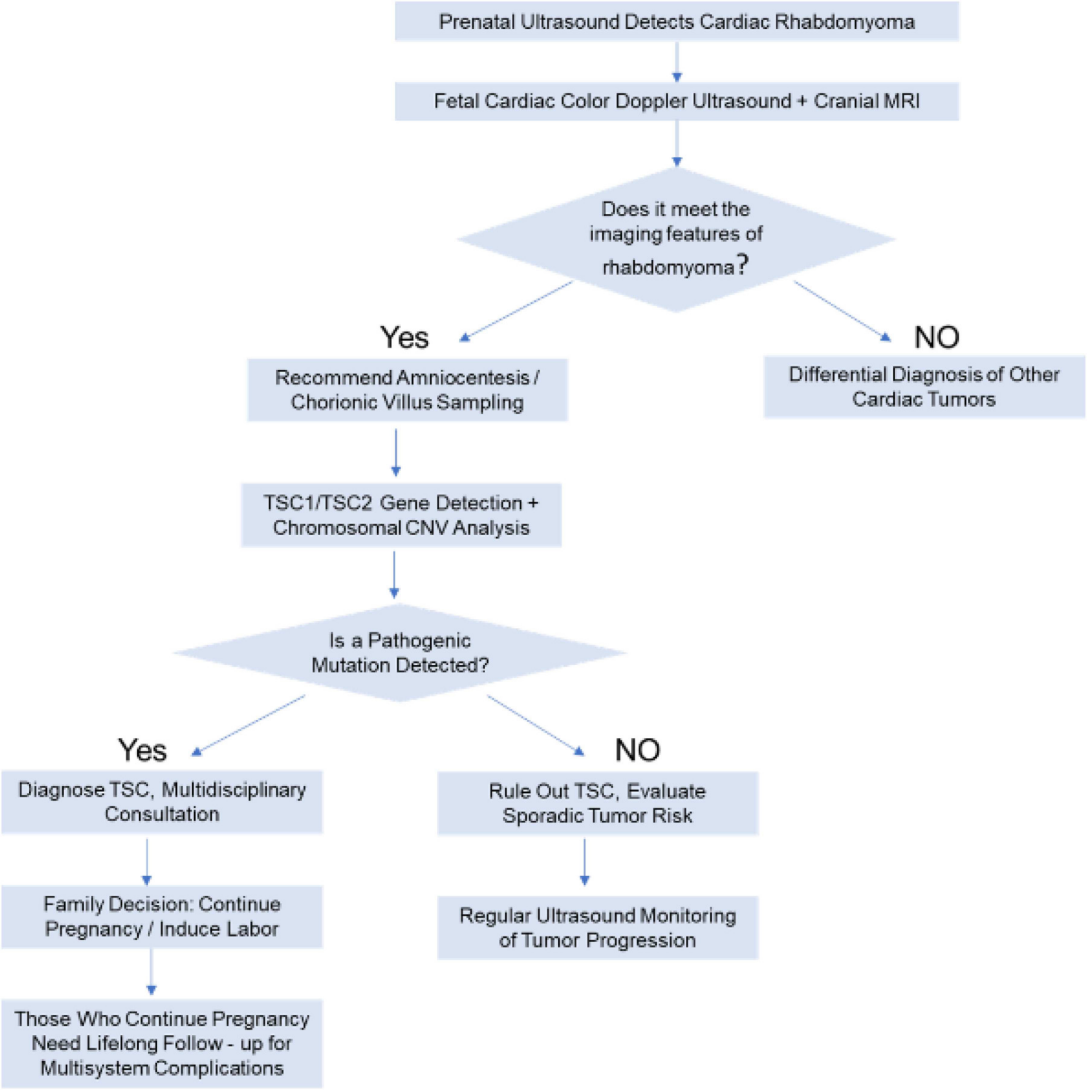
Management flowchart for prenatal detection of cardiac rhabdomyoma.

Prenatal diagnosis necessitates an integration of imaging studies, genetic testing, and clinical assessment. The specific approach is outlined below (Figure 3).

#### 3.2.1 Imaging studies

At approximately 20 weeks of gestation, fetal ultrasound may detect single or multiple hyperechoic nodules within the myocardium, which require differentiation from other cardiac tumors (e.g., fibroma, teratoma). Fetal echocardiography can further assess the tumor's location, size, and degree of blood flow obstruction. In cases where TSC is suspected, cranial magnetic resonance imaging (MRI) should be performed to screen for cortical tubers or subependymal giant cell astrocytomas (SEGAs), given that TSC may involve the central nervous system (9).

#### 3.2.2 Genetic testing

When cardiac rhabdomyoma is suggested by ultrasound, TSC1/TSC2 gene testing (e.g., whole-exome sequencing or targeted gene panels) is recommended, irrespective of family history (10). It is essential to determine whether the mutation is a pathogenic variant of TSC1 or TSC2 (e.g., nonsense, frameshift, or splice mutations) and to assess variant pathogenicity in accordance with ACMG guidelines (e.g., the TSC1 c.1498C>T nonsense mutation in this case was classified as likely pathogenic).

#### 3.2.3 Clinical assessment and family analysis

First, a multi-system involvement assessment should be performed. Beyond cardiac involvement, screening for other TSC-related manifestations in the fetus (e.g., renal hamartomas, skin lesions) should be conducted, although organ involvement may only manifest postnatally. Second, a family history should be obtained, including inquiries into whether parents and first-degree relatives have TSC-related symptoms (e.g., hypomelanotic macules, epilepsy, intellectual disability). Approximately 60% of TSC cases are familial, with 40% resulting from *de novo* mutations (11), consistent with the current case, where both parents had wild-type alleles and the fetus harbored a *de novo* mutation.

### 3.3 Characteristics of this case and clinical insights

In the present case, ultrasound findings suggested fetal cardiac rhabdomyoma, and when combined with the identification of a likely pathogenic TSC1 variant, a definitive diagnosis of Tuberous Sclerosis Complex type 1 (TSC1; OMIM: 191100) was established. The fetus exhibited only cardiac rhabdomyoma, with no evidence of brain nodules or skin lesions-consistent with the clinical heterogeneity of TSC. Furthermore, the TSC1 mutation in this case was a *de novo* variant, with neither parent harboring the variant-consistent with the observation that approximately 40% of TSC cases are sporadic. The mechanism underlying *de novo* mutations may involve errors in DNA replication or aberrations in epigenetic regulation during gametogenesis. Despite the parents’ normal phenotypes, germline mosaicism testing is warranted to rule out the extremely low risk of genetic transmission. Nevertheless, the overall risk of recurrent TSC in subsequent pregnancies is significantly lower than in familial cases (<1%) (12).

In this case, the patient declined prenatal genetic testing, and the diagnosis remained unconfirmed until after induced labor. This highlights the need to optimize the clinical management of fetal cardiac tumors: upon prenatal ultrasound detection of cardiac rhabdomyoma, amniocentesis should be performed promptly to analyze TSC1/TSC2 genes, and fetal cranial MRI should be recommended to screen for cortical tubers or subependymal giant cell astrocytomas (SEGAs) (13). If TSC is diagnosed prenatally, options such as continuing the pregnancy (with lifelong surveillance for multi-system complications) or undergoing induced labor can be pursued based on family preferences, thereby avoiding empiric decisions based solely on imaging findings.

Prenatal genetic testing (amniocentesis) was offered to the patient, but she declined due to concerns about procedure-related miscarriage risk (estimated at 0.5%–1% in our center). The decision for labor induction was based on a multidisciplinary team (MDT) assessment: while isolated cardiac rhabdomyomas may regress postnatally (14), the fetal echocardiogram showed multiple small rhabdomyomas (not isolated) and a family history of TSC (the patient's brother had TSC with severe neurological complications). These factors increased the suspicion of underlying TSC and potential long-term morbidity, leading the family to opt for termination after detailed MDT counseling.

In China, prenatal management of fetal cardiac rhabdomyomas follows a MDT approach (involving obstetrics, fetal medicine, genetics, and cardiology). Intrauterine medical treatment (e.g., everolimus) is currently in the exploratory stage in tertiary centers, with limited clinical data; thus, it is not yet a standard of care. Recent study (15) has reported successful treatment of fetal cardiac rhabdomyoma with mTOR inhibitors (everolimus or sirolimus) administered to the mother. For example, Maasz et al. observed a 30% reduction in fetal rhabdomyoma volume after 4 weeks of everolimus treatment (2.5 mg/day). However, such treatments carry potential risks, including maternal oral mucositis and fetal growth restriction, which require careful consideration in clinical decision-making.Spontaneous regression of isolated rhabdomyomas is recognized, and postnatal follow-up is recommended for such cases. Okutucu G et al. (2024) reported a case series of 9 patients with isolated fetal cardiac rhabdomyomas (no prenatal treatment) (14), with 6 cases showing spontaneous tumor size reduction by 6 months of age-consistent with the natural history of isolated rhabdomyomas.

### 3.4 Suggestions for future clinical management

Moving forward, efforts should be made to further standardize the clinical diagnosis and management of fetal cardiac rhabdomyoma (RM), with specific strategies outlined below:

#### 3.4.1 Optimizing clinical pathways

Upon detection of fetal cardiac rhabdomyoma via ultrasound, the prenatal diagnostic process should be initiated promptly, with clear communication to patients regarding TSC-associated risks and the necessity of genetic testing (6).

#### 3.4.2 Enhancing genetic counseling

Genetic counseling should be mandated as an integral component, utilizing visual aids, case illustrations, and other modalities to assist patients in understanding TSC's genetic basis, neonatal prognosis, and long-term management strategies (8).

#### 3.4.3 Establishing multidisciplinary teams

A multidisciplinary team (MDT) for fetal cardiac disorders should be established, comprising specialists from ultrasound, genetics, obstetrics, neonatology, and other relevant departments, to collaboratively develop diagnostic and intervention plans (e.g., in the current case, had the pregnancy continued, the neonatology team would have needed to preemptively plan postnatal cardiac and neurological monitoring) (16).

#### 3.4.4 Implementing precision genetic testing and risk assessment

For complex cases (e.g., *de novo* mutations or absence of family history), mutation types should be rapidly identified via gene panels or whole-exome sequencing, with assessment of parental germline mosaicism risk—though the probability is extremely low, this information should be communicated to families (17).

#### 3.4.5 Enhancing follow-up and reproductive counseling

For cases involving induced labor, genetic testing results should be promptly communicated post-induction, with reproductive counseling provided to parents (e.g., in the current case, the risk of recurrent TSC in subsequent pregnancies is <1%, with options including natural conception or assisted reproductive technologies) (18).

#### 3.4.6 Developing databases and evidence-based support

A TSC fetal management database should be established to track clinical outcomes associated with different mutation types, thereby providing an evidence base for managing subsequent cases.

## 4 Conclusion

When fetal cardiac rhabdomyoma is identified via prenatal ultrasound, TSC1/TSC2 gene testing should be incorporated into routine evaluations to differentiate hereditary TSC from sporadic cardiac tumors. The present case confirmed a *de novo* TSC1 mutation via whole-exome sequencing following induced labor, demonstrating that fetal cardiac rhabdomyoma can represent the initial manifestation of TSC even in the absence of a family history. Clinically, a standardized algorithm encompassing “ultrasound screening-genetic diagnosis-multidisciplinary consultation” should be implemented to deliver precise perinatal management and reproductive counseling to patients.

## Data Availability

The original contributions presented in the study are included in the article/supplementary material, further inquiries can be directed to the corresponding author/s.
